# Investigation of the relationship between food preferences and depression symptoms among undergraduate medical students: a cross-sectional study

**DOI:** 10.3389/fnut.2025.1519726

**Published:** 2025-03-10

**Authors:** Fatemeh Maleki Sedgi, Jalal Hejazi, Reza Derakhshi, Ghazal Baghdadi, Melinaz Zarmakhi, Mana Hamidi, Kamyar Mansori, Mohsen Dadashi, Mehran Rahimlou

**Affiliations:** ^1^Department of Nutrition, School of Public Health, Zanjan University of Medical Sciences, Zanjan, Iran; ^2^Social Determinants of Health Research Center, Health and Metabolic Diseases Research Institute, Zanjan University of Medical Sciences, Zanjan, Iran; ^3^Department of Epidemiology, School of Medicine, Zanjan University of Medical Sciences, Zanjan, Iran; ^4^Department of Clinical Psychology, School of Medicine, Shahid Beheshti Hospital, Zanjan University of Medical Sciences, Zanjan, Iran; ^5^Metabolic Diseases Research Center, Health and Metabolic Research Institute, Zanjan University of Medical Science, Zanjan, Iran

**Keywords:** food preferences, students, depression, cross-sectional, nutrition

## Abstract

**Introduction:**

Depression is a psychological condition characterized by a persistent low mood. This study investigates the relationship between depressive symptoms and food preferences in undergraduate students.

**Methods:**

A cross-sectional design was employed among 502 students at Zanjan University of Medical Sciences. Data collection included validated questionnaires on food preferences, depression levels (Beck Depression Inventory), and physical activity (IPAQ), along with anthropometric measurements. Food preferences were analyzed for six groups: grains, fruits, vegetables, dairy, meat/fish, and snacks. Depression severity was categorized into six levels based on BDI scores: normal (1–9), mild (10–16), borderline (17–20), moderate (21–30), severe (31–40), and very severe (41–63). Depression risk was defined as the odds of belonging to a higher Beck Depression Inventory (BDI) category.

**Results:**

Participants with severe depression had lower preferences for grains, fruits, and vegetables but higher preferences for snacks. Adjusted analyses revealed that higher preferences for fruits (OR: 0.79; 95% CI: 0.68–0.98) and vegetables (OR: 0.81; 95% CI: 0.71–0.94) were significantly associated with reduced depression risk, while snack preferences increased risk (OR: 1.28; 95% CI: 1.03–1.68). However, the association between grain preferences and depression risk was not statistically significant after adjustment (OR: 0.82; 95% CI: 0.74–1.03).

**Conclusion:**

These findings highlight the bidirectional link between diet and mental health, underscoring the importance of dietary interventions in mental health strategies.

## Introduction

Depression is one of the most well-known psychological conditions, characterized primarily by a persistent low mood. It is classified as a mental health disorder involving a mood disturbance, with common symptoms including feelings of sadness and unhappiness that can be temporary or long-lasting. The Diagnostic and Statistical Manual of Mental Disorders (DSM-IV) defines depression as a mood disorder characterized by a combination of cognitive, emotional, and physical symptoms that significantly impair daily functioning (1). Similarly, the Beck Depression Inventory (BDI), developed by Beck et al. (2), provides a widely accepted tool for assessing depression severity in clinical and research settings. The prevalence of depression has been increasing globally, particularly among young people, during critical periods of social, emotional, and cognitive development (3). For instance, rates of depression in the United States rose significantly between 2015 and 2019, especially among adolescents and young adults (4).

In Iran, recent estimates indicate a point prevalence of major depressive disorders of 4.8% in women and 2.3% in men, with a lifetime prevalence of 12.7% in the general population (5). Young adults in Iran face unique stressors, including academic pressure, financial challenges, and uncertainties about future career prospects, all of which may contribute to a rising burden of mental health disorders in this population (6). Understanding the prevalence and factors associated with depression in specific settings, such as Iran, is essential to developing targeted strategies for prevention and intervention.

Additionally, it is well-established that mood, emotions, and food choices are closely interrelated, creating a bidirectional relationship. Several mechanisms have been proposed to explain how food consumption and mood influence each other. Sensory experiences, such as taste and texture, can evoke immediate pleasure or discomfort, influencing mood (7). Cognitive expectations about a food’s effects, such as believing that chocolate can boost mood, can shape emotional responses to its consumption (8). Psychological distraction, where eating serves as a coping mechanism during stress or sadness, can temporarily alleviate negative emotions, reinforcing specific eating behaviors (9). Lastly, nutrients in foods directly modulate brain function by affecting neurotransmitter activity. For example, carbohydrates increase serotonin production, promoting a calming effect, while omega-3 fatty acids improve neuronal membrane function, which may enhance mood regulation. These mechanisms collectively underscore the complexity of the relationship between food preferences and mood, with no single mechanism exclusively accounting for observed patterns. Instead, they likely operate synergistically to shape dietary behaviors and emotional well-being (10).

Moreover, negative emotional states, such as those associated with depression, can lead to altered eating behaviors, including increased consumption of energy-dense, high-fat, and high-sugar foods, which are often perceived as comforting or rewarding (11, 12). In contrast, diets rich in whole grains, fruits, vegetables, and omega-3 fatty acids have been linked to improved mental health outcomes, suggesting that food choices can positively influence mood (13). Recent studies have highlighted a tendency toward sweet and high-calorie foods among individuals with depressive symptoms, emphasizing the complex interplay between diet and mental health (14, 15). Nutritional deficiencies, such as low levels of folate, magnesium, and omega-3 fatty acids, have been implicated in the pathophysiology of depression, further emphasizing the role of diet in mental health (16). This bidirectional relationship suggests that not only can depression shape dietary patterns, but dietary habits may also exacerbate or alleviate depressive symptoms.

Recent studies have increasingly emphasized the connection between nutrition and depressive disorders. A balanced intake of dietary nutrients has been associated with a reduced risk of developing depression (17–20). Quirk et al. (21) found that unhealthy dietary patterns, characterized by a high intake of processed foods, refined sugars, and saturated fats, were associated with an increased risk of depression, while diets rich in fruits, vegetables, whole grains, and omega-3 fatty acids were linked to lower depressive symptoms. These findings highlight the importance of evaluating the cumulative effects of dietary components rather than isolated nutrients (15, 21). This approach provides a more comprehensive understanding of how our eating habits influence mental health.

This study investigates the bidirectional relationship between depressive symptoms and food preferences among undergraduate students. We hypothesized that individuals with higher depressive symptoms would exhibit lower preferences for nutrient-dense foods (e.g., fruits, vegetables, and grains) and higher preferences for energy-dense, low-nutrient foods (e.g., snacks). Furthermore, we anticipated that higher preferences for nutrient-dense foods would be associated with reduced odds of being classified into higher depressive symptom categories, while preferences for low-nutrient foods would increase these odds. In this study, depressive symptoms serve as the dependent variable, while food preferences are considered the independent variable. Understanding this bidirectional relationship could offer valuable insights for developing tailored dietary and mental health strategies for young adults in academic settings.

## Method

### Study design and participant recruitment

This cross-sectional study was conducted at Zanjan University of Medical Sciences from December 2023 to June 2024. A total of 502 undergraduate students from diverse academic fields, including Medicine, Paramedicine, Public Health, Nursing, and Midwifery, were recruited using convenience sampling. Participants eligible for the study were undergraduate students aged 18 years or older who consented to participate voluntarily. Individuals were excluded if they were pregnant, lactating, diagnosed with chronic diseases (e.g., diabetes, hypertension, and cancer), autoimmune conditions (e.g., rheumatoid arthritis), or following specific dietary regimens (e.g., vegetarian or weight-loss diets). Additionally, individuals diagnosed with cancer, rheumatoid arthritis, autoimmune diseases, severe inflammatory conditions, or other chronic illnesses such as cardiovascular disease, chronic kidney disease, or diabetes were excluded. A complete case analysis was used to ensure that only participants with fully available data were included in the final analysis.

The sample size was calculated based on a study by Shahani-Yeilaghi and Basaknejad (22), considering the criteria for food addiction, with a 95% confidence interval and 80% power. The calculated sample size was 462 participants, which was increased to 500 to account for an estimated 10% of missing or unusable data.


$$n=\frac{4\times {\left(z_{\frac{\alpha }{2}}+z_{\beta }\right)}^{2}}{{\left[Ln\left(\frac{1+r}{1-r}\right)\right]}^{2}}+3$$

$$n=\frac{4\times {\left(1.96+0.84\right)}^{2}}{{\left[Ln\left(\frac{1+0.12}{1-0.12}\right)\right]}^{2}}+3=462$$

Convenience sampling was employed to recruit participants. At dormitories, three trained interviewers approached students in common areas such as lounges and study rooms during designated times when students were likely to be present. Off-campus participants were recruited by contacting students listed in the university’s extracurricular activity groups and inviting them to participate in the study. All participants were provided with a brief explanation of the study and were invited to fill out the questionnaires voluntarily.

Participants were informed about the general purpose of the study, which was to investigate the relationship between food preferences and depressive symptoms among students. Detailed explanations regarding study objectives, procedures, and the voluntary nature of participation were provided before obtaining written informed consent. Care was taken not to disclose hypotheses that might bias participant responses. Demographic data were collected using a self-designed questionnaire that included information on age, marital status, education level, academic field and medication use.

### Ethics approval and consent to participate

This study was approved by the Ethics Committee of Zanjan University of Medical Sciences (IR.ZUMS.REC.1402.010). All participants provided written informed consent after being fully informed about the study’s objectives, procedures, potential risks, and benefits. Participants were not remunerated for their participation in the study.

### Food preferences questionnaire

The Food Preferences Questionnaire (FPQ) was used to assess participants’ preferences for six food categories: vegetables, fruits, meat/fish, dairy, snacks, and starches. The FPQ developed by Smith et al. (23) was utilized to assess food preferences in six categories: vegetables, fruits, meat/fish, dairy, snacks, and starches. The adapted Persian version of the FPQ consisted of 62 items, reflecting modifications for cultural relevance and in 2024, its validity and reliability were confirmed among Iranian youth (24). Specifically, 12 items from the original FPQ were removed because they were not commonly consumed in Iran (e.g., bacon and other pork-based items), and 15 items were added to reflect foods frequently consumed in Iran. Examples include Iranian cheeses (e.g., Feta, Lighvan, and Tabriz), locally consumed fruits (e.g., pomegranate and quince), vegetables (e.g., fresh herbs and eggplant), and traditional snacks (e.g., nuts and dried fruits). The questionnaire was administered using a 6-point Likert scale ranging from “strongly dislike” (score: 1) to “strongly like” (score: 5), with an additional option for “have not tried/do not know” (excluded from scoring).

The six subcategory scores were calculated as the sum of individual item scores within each category. The total FPQ score was computed as the sum of the six subcategory scores, with higher scores indicating a stronger preference for a broader variety of foods. FPQ scores were analyzed both continuously and categorically. For categorical analysis, quartiles were generated based on the FPQ total score distribution in the sample. These quartiles represented increasing levels of food preferences, and associations with depression risk were examined accordingly. Additional quartiles were generated separately for each subcategory score to assess category-specific associations.

The validity and reliability of this adapted Persian version of the FPQ were confirmed by Mohammadifard et al. (54) demonstrating high consistency for use among young individuals. In this study, we employed this validated Persian version of the FPQ to ensure the cultural relevance and accuracy of assessing food preferences among our participants.

### Depression assessment

Depression severity was assessed using the Beck Depression Inventory (BDI) (25), a validated tool for measuring depressive symptoms. The BDI includes 21 items, each scored on a 4-point Likert scale ranging from 0 (“not at all”) to 3 (“severe”). Total scores range from 0 to 63, with higher scores indicating greater severity of depressive symptoms. Based on the original BDI scoring protocol, participants were categorized into six groups: 1–9 (normal), 10–16 (mild depression), 17–20 (borderline depression), 21–30 (moderate depression), 31–40 (severe depression), and 41–63 (very severe depression) (26, 27). For the purposes of this study, “depression risk” was operationally defined as having a BDI score greater than 9 (i.e., falling into any category other than “normal”). This threshold was used to dichotomize the sample for certain analyses. However, to provide a more nuanced understanding, depression categories were also analyzed individually using ordinal logistic regression, where appropriate, and compared across groups using ANOVA. The choice of this cut-off aligns with established conventions in psychological research and clinical practice, ensuring both rigor and comparability with existing studies. To further contextualize these choices, references to the original scoring protocol (26) and the validation studies in similar populations (27) have been included.

In Persian settings, the BDI has been translated, culturally adapted, and validated in prior studies, showing good reliability and construct validity (Cronbach’s alpha ≥0.8) (28). Despite its robustness, cultural factors in Iran may influence how depressive symptoms are expressed and reported. Iranian culture often emphasizes somatic symptoms (e.g., fatigue, headaches) over emotional symptoms due to social norms and stigma surrounding mental health, which may impact the interpretation of certain BDI items. Additionally, the stigma associated with mental illness may lead to underreporting of symptoms, especially in younger populations. These considerations are important when interpreting the study’s results.

### Anthropometric measurements

Anthropometric measurements, including height, weight, and waist and hip circumferences, were taken by trained staff. All staff members received standardized training on measurement protocols to ensure consistency and accuracy. Height was measured using a stadiometer (Seca 213, Germany), and weight was recorded using a calibrated digital scale (Seca 813, Germany). Waist and hip circumferences were measured using a non-elastic measuring tape (Gulick II, Country Technology, United States).

Each measurement was taken twice, and the average of the two measurements was used for analysis. If discrepancies between the two measurements exceeded (more than 0.5 cm), a third measurement was taken, and the two closest values were averaged. Body measurements, including weight, height, and waist and hip circumferences, were taken using standardized equipment. Body mass index (BMI) was calculated as weight (kg) divided by height squared (m^2^).

### Physical activity assessment

Physical activity levels were assessed using the short form of the International Physical Activity Questionnaire (IPAQ-SF), which consists of seven questions evaluating the frequency and duration of walking, moderate-intensity activities, and vigorous-intensity activities over the past 7 days. The IPAQ-SF has been validated in various populations, including Iranian adults, demonstrating acceptable reliability and validity (29). The validity and reliability of this tool have previously been assessed in Iranian adult women and were found to be satisfactory (30). For data analysis, physical activity levels were quantified in Metabolic Equivalent Task (MET) minutes per week (MET-min/week). The total MET score was calculated using the following formula:

Walking: MET-min/week = Walking minutes × days × 3.3 METs.

Moderate activity: MET-min/week = Moderate activity minutes × days × 4.0 METs.

Vigorous activity: MET-min/week = Vigorous activity minutes × days × 8.0 METs.

Participants were then categorized into three physical activity levels based on the IPAQ scoring protocol:

Low: <600 MET-min/week.

Moderate: 600–2,999 MET-min/week.

High: ≥3,000 MET-min/week.

These categorical classifications were used in Table 1 to summarize participants’ physical activity levels, while continuous MET scores were used in further statistical analyses.

**Table 1:** Basic characteristics and depression status of participants.

Variables	Mean/Frequency
Age (Years)	22.09 ± 3.15
Weight (kg)	68.58 ± 14.50
Height (cm)	171.19 ± 9.47
BMI (kg/m^2^)	23.28 ± 3.88
BDI depression score	15.01 ± 7.77
Gender	
Male (%)	283(57%)
Female (%)	219 (43%)
Number of students by field	
Nutritional Sciences	73(14.54)
Nursing	148(29.28)
Health-related fields	176(35.09)
Midwifery	41(8.16)
Paramedical fields	64(12.74)
Physical activity	
Low	112(22.5)
Moderate	244(48.7)
High	144(28.8)
Smokers	43(8.7)
Depression status	
No depression	326(65%)
Mild depression	115(23%)
Moderate depression	48(10%)
Severe depression	13(2%)

Standard deviation. Physical activity: Low: <600 MET-min/week, Moderate: 600–2,999 MET-min/week, High: ≥3,000 MET-min/week.

### Covariates

Several covariates were included in the analysis to account for potential confounders. Age was self-reported by participants in full years at the time of the study. Gender was assessed through self-report and categorized as male or female. In this study, we did not assess gender identity beyond these binary categories. Smoking was assessed using a self-administered questionnaire. Participants were asked whether they had ever smoked (yes/no) and whether they were currently smoking (yes/no). Pack years or other quantitative measures of smoking were not collected. Physical activity was measured using the IPAQ short form, which categorizes participants into three levels: low, moderate, or high physical activity, based on the frequency and intensity of activity reported over the past week. BMI was calculated as weight (kg) divided by height (m^2^), using anthropometric measurements taken by trained staff (as detailed in the “Anthropometric Measurements” subsection). Participants reported their academic field of study, which was categorized into nutritional sciences, nursing, health-related fields, midwifery, or paramedical fields.

### Data analysis

In this study, the primary direction of analysis was to investigate the relationship between food preferences (independent variable) and depressive symptoms (dependent variable). Depressive symptoms were measured using the BDI, while food preferences were assessed using the validated Persian version of the FPQ. In total, the survey consisted of 90 questions across the three instruments (62 items for FPQ, 21 items for BDI and 7 items for IPAQ). On average, participants required approximately 20–25 min to complete the survey, including time to review the instructions. Trained interviewers were present during the survey administration to provide guidance and answer any questions participants had during the process, ensuring accurate and complete responses.

After collecting all the questionnaires and reaching the required sample size, the data were entered into SPSS version 22 for analysis. The normality of the data distribution within the six food preference categories and the overall food preference score was assessed using the Kolmogorov–Smirnov test, confirming that the data were normally distributed.

For analysis, depression severity was examined both as a categorical variable (using the six predefined categories) and as a continuous variable (total BDI score). “Depression risk,” as reported in the abstract, refers to participants with BDI scores of 9 or higher, encompassing all categories from “mild depression” to “very severe depression.” This threshold is based on the BDI scoring guidelines, which define scores of 10 or higher as indicative of clinically significant depressive symptoms.

Pearson’s correlation test was utilized to analyze the relationships between continuous variables. Independent t-tests were employed to compare dietary intake between male and female students, and ANOVA was used to examine differences in food preference scores across varying levels of depression severity. To evaluate the association between food preference scores and depression risk, logistic regression models were employed. Three models were used to adjust for potential confounders incrementally: model 1 (crude), this model included no covariates and examined the unadjusted association between food preference scores (total and by food group) and depression risk. Model 2 (Partially Adjusted): This model adjusted for age and physical activity as these variables are well-established confounders in studies examining depression and dietary behaviors. Age was self-reported in years, and physical activity was assessed using the IPAQ, as described in the “Covariates” section. Model 3 (Fully Adjusted): This model further adjusted for smoking status, BMI, and gender in addition to the variables in Model 2. Smoking was included because it has been linked to both dietary habits and depression risk. BMI was added as a measure of overall health and nutrition status, and gender was included to account for potential gender-based differences in food preferences and mental health outcomes. The stepwise approach was used to evaluate the individual and combined effects of these covariates on the observed associations. Covariates were selected based on their theoretical relevance, evidence from prior literature, and availability in the dataset. Statistical significance was set at *α* = 0.05, and *p*-values were considered statistically significant if *p* < 0.05.

## Results

### Participant characteristics

In terms of the baseline characteristics of the 502 participants (Table 1), the average age was 22.09 ± 3.15 years, and the mean depression questionnaire score was 15.01 ± 7.77. The average BMI was calculated as 23.28 ± 3.88. The majority of participants were male 283 (57%), with a significant portion studying health-related fields 176 (35.09%). Regarding depression status (Table 1), 326 participants (65%) showed no signs of depression, 115 (23%) experienced mild depression, 48 (10%) had moderate depression, and 13 (2%) had severe depression.

Additionally, Table 2 shows the mean Food Preference Scores among the participants. The overall mean score derived from the 62 questions on food preferences among the participants was (330.26 ± 45.78). When comparing the total food preference scores between male and female students, no significant difference was observed (*p* = 0.67). Table 2 highlights a significant difference (*p* < 0.001) in meat group preference scores between male students (48.14 ± 7.92) and female students (44.54 ± 8.20). However, there were no significant differences observed between male and female students in terms of other food groups.

**Table 2:** Mean food preference scores among participants.

Variable	Total score Mean ± SD	Male students Mean ± SD	Female students Mean ± SD	*P*-value^1^
Total food preference score	330.26 ± 45.78	333.36 ± 49.97	328.2 ± 42.76	0.672
Meat group score	45.98 ± 8.26	48.14 ± 7.92	44.54 ± 8.20	<0.001
Dairy group score	32.45 ± 7.01	32.80 ± 7.34	32.22 ± 6.79	0.463
Grains group score	47.40 ± 8.06	46.40 ± 8.54	47.13 ± 8.13	0.441
Fruits group score	91.61 ± 15.15	92.32 ± 16.98	91.14 ± 13.83	0.593
Vegetables group score	73.70 ± 16.29	74.16 ± 17.27	73.39 ± 15.66	0.665
Snacks group score	37.69 ± 6.58	37.48 ± 6.79	37.84 ± 6.44	0.776

^1Calculated by independent sample t-test, Data reported as mean ± SD.^

### Relationship between food preferences and depression risk

Table 3 shows that as the severity of depression increased, the mean total food preference score decreased. For example, participants without depression had the highest scores, while those with severe depression exhibited significantly lower scores, indicating a strong inverse relationship between depression severity and food preference. Participants with severe depression showed the lowest preference scores for nutrient-dense food groups such as grains, fruits, and vegetables (Table 3). This suggests that depressive symptoms may drive a preference for less healthful food choices, which could have implications for nutritional interventions in this population. Conversely, those with severe depression had the highest food preference score for snacks (39.71 ± 9.41), with a significance level of (*p* = 0.034) when compared to other groups, while those without depression had the lowest score (35.90 ± 6.60). However, regarding the meat and dairy groups, the results did not show significant differences in the food preference scores (*p* > 0.05).

**Table 3:** The mean food preference scores across different food groups.

Depression severity	Total score	Meat group	Dairy group	Grains group	Fruits group	Vegetables group	Snacks group
No depression	335.90 ± 44.79	46.19 ± 8.29	32.71 ± 7.06	47.40 ± 8.06	93.55 ± 18.55	76.18 ± 15.45	35.90 ± 6.60
Mild depression	329.57 ± 45.57	47.32 ± 7.45	32.51 ± 7.36	46.64 ± 8.74	89.56 ± 15.59	72.85 ± 16.27	36.39 ± 5.75
Moderate depression	317.57 ± 43.14	45.73 ± 8.78	31.60 ± 6.34	43.98 ± 8.12	87.09 ± 45.13	68.86 ± 15.88	38.67 ± 6.99
Severe depression	302.57 ± 70.13	45.28 ± 9.37	32.57 ± 8.28	42.01 ± 7.19	85.42 ± 19.27	60.85 ± 26.90	39.71 ± 9.41
*P*-value^1^	<0.001	0.065	0.214	<0.001	<0.001	<0.001	0.034

^1Calculated by ANOVA.^

Table 4 presents the odds ratios (OR) for the association between food preference scores and depression risk, comparing the highest quartile (Q4) to the lowest quartile (Q1) of food preference scores. The crude analysis revealed that having a higher total Food Preference Score was significantly associated with a lower risk of depression (OR: 0.68, 95% CI: 0.52–0.81, *p* < 0.01). This association remained significant even after adjusting for age and physical activity (OR: 0.69, 95% CI: 0.52–0.83), age, physical activity, and smoking (OR: 0.72, 95% CI: 0.58–0.87), and age, physical activity, smoking, BMI, and gender (OR: 0.79, 95% CI: 0.68–0.91).

**Table 4:** Association between food preference scores and the risk of depression.

Variable (Q4 vs. Q1)^1^	Crude OR (95% CI)	Adjusted OR for age and physical activity (95% CI)	Adjusted OR for age, physical activity, and smoking (95% CI)	Adjusted OR for age, physical activity, smoking, BMI, and gender (95% CI)
Total food preference score	0.68 (0.52–0.81) ^**^	0.69 (0.52–0.83) ^**^	0.72 (0.58–0.87) ^**^	0.79 (0.68–0.91) ^*^
Meat group score	0.91 (0.83–1.08)	0.92 (0.86–1.09)	1.03 (0.94–1.12)	1.06 (0.98–1.14)
Dairy group score	0.88 (0.78–1.03)	0.89 (0.78–1.05)	0.95 (0.85–1.10)	0.98 (0.88–1.12)
Grains group score	0.70 (0.61–0.87) ^***^	0.75 (0.64–0.90) ^**^	0.78 (0.66–0.97) ^*^	0.82 (0.74–1.03)
Fruits group score	0.63 (0.55–0.82) ^***^	0.68 (0.59–0.84) ^***^	0.73 (0.65–0.89) ^**^	0.79 (0.68–0.98) ^*^
Vegetables group score	0.72 (0.58–0.82) ^***^	0.73 (0.60–0.83) ^**^	0.75 (0.67–0.86) ^**^	0.81 (0.71–0.94) ^*^
Snacks group score	1.20 (1.04–1.39) ^**^	1.43 (1.05–1.91) ^**^	1.22 (1.00–1.48) ^*^	1.28 (1.03–1.68) ^*^

OR; Odds Ratio, CI; Confidence Interval, * indicates a significance level of less than 0.05, ** indicates a significance level of less than 0.01, *** indicates a significance level of less than 0.001, ^1^Logistic regression models were used in the analysis. Model 1: Crude analysis with no covariate adjustment. Model 2: Adjusted for age and physical activity. Model 3: Fully adjusted for age, physical activity, smoking, BMI, and gender.

Regarding specific food groups, the grains group showed a significant inverse association with depression risk in the crude model (OR: 0.70, 95% CI: 0.61–0.87, *p* < 0.001), which persisted after adjusting for multiple factors. Similarly, the fruits group was associated with a reduced risk of depression (crude OR: 0.63, 95% CI: 0.55–0.82, *p* < 0.001) and maintained its significance after all adjustments. The vegetables group also showed a consistent inverse relationship with depression risk across all models (crude OR: 0.72, 95% CI: 0.58–0.82, *p* < 0.001).

In contrast, no significant association was observed between the meat and dairy groups and depression risk in any of the models (*p* > 0.05). Interestingly, the snacks group showed a positive association with depression risk, with significantly higher odds of depression in the highest quartile of preference (crude OR: 1.20, 95% CI: 1.04–1.39, *p* < 0.01), which remained significant after all adjustments. These results suggest that higher preferences for grains, fruits, and vegetables are associated with a reduced risk of depression, while a preference for snacks is associated with an increased risk of depression.

### Depression symptoms and food preferences

To examine the bidirectional relationship, we investigated how depressive symptoms influenced food preferences by comparing mean food preference scores across different depression severity levels (Table 3). Results indicated that individuals with more severe depressive symptoms exhibited significantly lower preferences for total food (Mean: 302.57, SD: 70.13), grains (Mean: 42.01, SD: 7.19, *p* < 0.001), fruits (Mean: 85.42, SD: 19.27, *p* < 0.001), and vegetables (Mean: 60.85, SD: 26.90, *p* < 0.001) compared to those with mild or no depression.

Conversely, individuals with severe depression showed the highest preference for snacks (Mean: 39.71, SD: 9.41, *p* = 0.034) compared to those with mild or no depression (Mean for no depression: 35.90, SD: 6.60). However, no significant differences were observed for meat (*p* = 0.065) and dairy (*p* = 0.214) preferences across depression severity levels.

These results highlight statistically significant differences in food preferences across depression severity levels, with notable variations in snack consumption and decreased preferences for nutrient-dense food categories among individuals with higher depressive symptoms.

## Discussion

Our study investigated the bidirectional relationship between depressive symptoms and food preferences among undergraduate students. As hypothesized, individuals with higher depressive symptoms exhibited lower preferences for nutrient-dense foods (e.g., fruits, vegetables, and grains) and higher preferences for energy-dense, low-nutrient foods (e.g., snacks). Additionally, we hypothesized that higher preferences for nutrient-dense foods would be associated with reduced odds of being classified into higher depressive symptom categories, while preferences for low-nutrient foods would increase these odds. Our findings largely support these hypotheses, as higher fruit and vegetable preferences were significantly associated with lower depression risk, while snack preferences were linked to an increased risk. However, while the crude analysis suggested an inverse association between grain preferences and depression risk, this relationship was not statistically significant after full adjustment, suggesting potential confounding effects.

This finding supports previous evidence suggesting a bidirectional relationship between mood and dietary choices. Emotional states can influence food preferences, while dietary habits may also contribute to mental health outcomes (31). Various psychological and biological mechanisms may explain this relationship, including emotional eating, neurotransmitter activity, and stress-related hormonal responses (32).

However, contrary to our results, Haghighat et al. (33) reported no significant relationship between depression and general food cravings (34). Nonetheless, they identified a specific positive association between depression and craving for jelly, a snack with a high glycemic index. This suggests that while general food preferences might not always align with depressive states, certain high-sugar foods could still be more appealing to those experiencing depressive symptoms. Furthermore, longitudinal studies have demonstrated that the consumption of sugary drinks, refined foods, fried foods, processed meats, refined grains, high-fat diets, as well as snacks like biscuits and pastries, is linked to an increased risk of developing depression (35, 36). Rahimlou et al. (37), in a systematic review, found that the consumption of lower glycemic index (GI) carbohydrates is associated with a decreased risk of depression.

To understand the underlying mechanisms behind these associations, it is essential to consider psychological, biological, and hormonal factors. Serotonin, a key neurotransmitter involved in mood regulation, has been shown to influence food preferences. In human studies, it was found that carbohydrate intake stimulates insulin release, which increases the ratio of tryptophan in the brain, ultimately enhancing serotonin synthesis (38). This may explain why individuals with depressive symptoms often gravitate toward carbohydrate-rich snack foods, as a form of self-regulation to improve mood.

Additionally, the dopamine reward system, which is often dysregulated in depression, plays a crucial role in emotional eating. Depressed individuals may experience reduced dopamine activity, leading to cravings for energy-dense, palatable foods that provide short-term reward and relief (39). Finally, elevated cortisol levels in depression, driven by chronic stress and hypothalamic–pituitary–adrenal (HPA) axis dysregulation, can increase preferences for high-fat and sugary foods, further contributing to unhealthy dietary patterns (40). In individuals with depression, dysregulation of the dopaminergic system has been implicated in altered eating behaviors, particularly emotional eating and restricted eating. Emotional eating, which refers to consuming food in response to negative emotions rather than hunger, is often driven by the brain’s reward system. Dopamine, a key neurotransmitter involved in pleasure and reward, plays a crucial role in this mechanism (41).

Research suggests that individuals with depression experience blunted dopamine activity, which may lead to compensatory overeating of palatable foods (high in sugar and fat) to stimulate dopamine release (42). However, it is important to note that this is just one potential pathway linking depression to food preferences. Other factors, such as serotonin regulation, gut microbiota composition, and inflammatory processes, also contribute to altered dietary choices. Additionally, restricted eating patterns, commonly seen in depression, may further exacerbate food preference changes by increasing cravings for energy-dense foods. Studies have shown that individuals with depression tend to have a higher preference for processed snacks and sweets while consuming fewer nutrient-dense foods like fruits and vegetables, reinforcing the bidirectional relationship between diet and mood (43).

In individuals with major depression, evidence indicates that the activation of the immune system results in an overproduction of pro-inflammatory immune cells, originating from both the peripheral system (such as macrophages) and the brain (including microglia and astrocytes). This excessive immune response triggers a cascade of inflammatory changes within the brain, which plays a significant role in the development of depressive symptoms (44). Pro-inflammatory cytokines can lead to various symptoms, such as anorexia, loss of libido, sleep disturbances, impaired short-term memory, and heightened sensitivity to pain (hyperalgesia). Additionally, these cytokines can activate the hypothalamic–pituitary–adrenal (HPA) axis by stimulating central corticotropin-releasing factor (CRF) neurons, ultimately resulting in elevated cortisol levels (hypercortisolemia) (45).

Clinical evidence further suggests that the chronic hypersecretion of cortisol, a common characteristic of depression, causes metabolic changes like increased abdominal fat and bone decalcification (46). Over time, this sustained elevation in cortisol levels leads to the desensitization of glucocorticoid receptors in both the brain and immune cells, diminishing their responsiveness to cortisol and exacerbating the body’s stress response.

As previously mentioned, there appears to be a significant link between inflammation and depression. Our findings indicate that individuals with depression showed a lower preference for consuming vegetables, fruits, and grains. These food groups are known to be rich sources of B vitamins (such as B6, B12, and folate), fiber, magnesium, and various antioxidants (47).

Recent studies have highlighted that deficiencies in B vitamins, particularly folate, are common among individuals with depression (48). Several mechanisms may explain the connection between low folate levels and increased depression risk. These mechanisms include reduced neurotransmitter synthesis, impaired methylation processes leading to decreased levels of *S*-adenosyl methionine (SAMe) and elevated homocysteine levels, as well as direct effects on the central nervous system (49, 50).

Moreover, a low intake of dietary magnesium, which is found in foods such as vegetables, nuts, seeds, fish, legumes, and whole grains, has also been associated with a higher risk of depression (49).

Dietary fiber from fruits and vegetables plays a crucial role in shaping the gut microbiota composition, which in turn influences mood and depression through the gut-brain axis. The gut-brain axis is a bidirectional communication system linking the central nervous system and the gastrointestinal tract via neural, hormonal, and immune pathways. A diet rich in fiber promotes the growth of beneficial gut bacteria, such as Bifidobacterium and Lactobacillus species, which produce short-chain fatty acids (SCFAs) like butyrate, acetate, and propionate. These SCFAs have anti-inflammatory properties and can modulate brain function by reducing neuroinflammation and enhancing the integrity of the blood–brain barrier (51).

Conversely, diets high in processed foods and low in fiber may lead to dysbiosis, an imbalance in the gut microbiota, characterized by a reduction in beneficial bacteria and an overgrowth of pathogenic species. Dysbiosis is associated with increased intestinal permeability (“leaky gut”), which allows inflammatory molecules such as lipopolysaccharides (LPS) to enter systemic circulation, triggering inflammation that affects brain function and mood regulation (52). Our findings, which show a lower preference for fruits and vegetables among individuals with depressive symptoms, suggest that such dietary patterns may exacerbate dysbiosis and contribute to depression via these mechanisms.

The antioxidants found in fruits and vegetables, such as vitamin C, vitamin E, and polyphenols, provide protective effects against depression by neutralizing oxidative stress. Oxidative stress occurs when there is an imbalance between the production of reactive oxygen species (ROS) and the body’s ability to counteract their harmful effects with antioxidants. Excess ROS can damage lipids, proteins, and DNA, contributing to chronic low-grade inflammation. This inflammation is a well-documented factor in the pathophysiology of depression, as it disrupts neurochemical signaling and promotes neuronal damage (53). By scavenging ROS and reducing oxidative damage, dietary antioxidants help mitigate inflammation and protect against the neuroinflammatory processes linked to depression (53).

One of the strengths of our study is its focus on the relationship between depression and food preferences in a student population, providing valuable insights into the bidirectional impact of diet and mental health. Additionally, the use of validated questionnaires for assessing both depression and dietary habits enhances the reliability of our results.

This study has several limitations. First, the self-reported nature of food preference and depression questionnaires may have introduced recall or social desirability bias. Second, informing participants about the study’s purpose might have influenced their responses, particularly if they adjusted their answers to align with perceived expectations. Furthermore, social desirability bias could have influenced responses, as participants may have reported healthier dietary habits or minimized symptoms of depression to align with perceived expectations. Future studies could minimize this bias by using a more blinded or indirect approach to assessing these variables. Third, although the Persian version of the BDI has been validated for use in Iranian populations, cultural differences in the perception and expression of depressive symptoms may have influenced our findings. For example, Iranian individuals may place greater emphasis on somatic rather than emotional symptoms, which could affect responses to specific items on the BDI. Four, mental health stigma may lead to underreporting of depressive symptoms, particularly among students. Future research could address these limitations by incorporating alternative culturally specific diagnostic tools or qualitative methodologies to capture the nuances of depression in Persian contexts.

Finally, a key limitation of our study is that the participant population consisted exclusively of undergraduate students in health sciences fields. This may limit the generalizability of our findings to the broader student population, as health sciences students might have greater awareness of nutrition and mental health, potentially influencing their dietary habits and attitudes toward depression. Additionally, their academic environment, which includes exposure to health-related education, may affect their food choices differently than students from other disciplines. Future research should consider including students from diverse academic backgrounds to improve the external validity of these findings.

These results have important public health and policy implications. Universities and health organizations should consider integrating nutrition education into mental health initiatives, emphasizing the role of nutrient-dense foods in psychological well-being. Campus-wide interventions could include improving access to affordable, healthy food options, offering dietary counseling for students at risk of depression, and implementing stress-management programs that incorporate nutritional guidance.

Future research should explore the causal pathways between diet and depression, particularly through longitudinal studies and randomized controlled trials. Examining how dietary interventions influence mental health outcomes will be essential in developing evidence-based recommendations. Additionally, expanding this research to include students from diverse academic backgrounds and socioeconomic groups will improve the generalizability of these findings. Given the bidirectional relationship between diet and mental health, addressing both dietary habits and psychological well-being in young adults should be a priority for future mental health strategies.

## Conclusion

In conclusion, our study highlights a bidirectional relationship between depressive symptoms and food preferences among undergraduate students. We found that individuals with higher depression severity exhibited lower preferences for nutrient-dense foods, including grains, fruits, and vegetables, while showing a stronger preference for snacks. This suggests that depressive symptoms may drive unhealthy dietary choices, potentially exacerbating mental health challenges.

Conversely, we also found that food preferences were significantly associated with depression risk. Higher preferences for fruits and vegetables were linked to lower odds of depression, while snack consumption was associated with an increased risk. However, the association between grain preferences and depression risk was attenuated after full adjustment, suggesting potential confounding factors. These findings emphasize the importance of considering both dietary habits and mental health status when developing interventions to support student well-being.

## Data Availability

The raw data supporting the conclusions of this article will be made available by the authors, without undue reservation.
